# Hydrogeochemical and biomedical insights into germanium potential of curative waters: a case study of health resorts in the Sudetes Mountains (Poland)

**DOI:** 10.1007/s10653-017-0061-0

**Published:** 2018-01-03

**Authors:** Dariusz Dobrzyński, Anna Boguszewska-Czubara, Kenji Sugimori

**Affiliations:** 1 Department of Hydrogeochemistry and Groundwater Dynamics, Faculty of Geology, University of Warsaw, Zwirki i Wigury 93, 02-089 Warsaw, Poland; 2 Department of Medical Chemistry, I Faculty of Medicine, Medical University of Lublin, Chodźki 4a, 20-093 Lublin, Poland; 3 Department of Biology, Toho University Faculty of Medicine, 5-21-16, Oomori-nishi, Oota-ku, Tokyo 143-8540 Japan

**Keywords:** Germanium geochemistry, Curative water, Mineral water, Therapeutic use, Germanium metabolism, Poland

## Abstract

Germanium is considered to be a non-essential element; however, little is still known about its significance for living organisms. It exerts prophylactic and therapeutic effects in the treatment of serious diseases such as cancer, HIV infection, and others. Germanium does not exhibit acute toxicity, but, as it tends to accumulate in various organs and tissues, undesirable and even dangerous side effects have been reported after prolonged and/or high dosage application. In general, inorganic compounds of germanium are more toxic than its organic compounds. Further studies should be performed to elucidate the exact molecular mechanism of germanium action, to determine the safe and effective dose of germanium via curative/mineral waters, and to understand the applications and benefits of using germanium-enriched waters in balneotherapy. The geochemistry of curative (cold CO_2_-rich, thermal) waters from spas in the Sudetes (Poland) was clarified in terms of components and mineral phases which might govern germanium. Germanium and silicon in thermal (above 20 °C) waters presumably result from the solubility of silicates in crystalline (granites, gneisses) aquifer rocks and might be controlled by neo-formed quartz. The cold CO_2_-rich waters revealed a significant diversity of aqueous chemistry and relationships of germanium with iron, silicon, or arsenic. Locally, both in sedimentary (sandstones) and metamorphic (gneisses) aquifer rocks, primary (silicates) and/or secondary (oxides) iron-containing minerals likely release germanium into solution. In the CO_2_-rich waters of the western part of the Kłodzko Region, germanium distinctly correlates with arsenic. It is hypothesized that both elements are co-sourced from crystalline basement and/or migration of substances of post-magmatic origin along deep-seated dislocations related to the seismically active Poříčí-Hronov fault zone. This area was proposed as the most prospective one for finding waters rich in germanium in the Sudetes.

**Electronic supplementary material:**

The online version of this article (10.1007/s10653-017-0061-0) contains supplementary material, which is available to authorized users.

## Introduction

Although germanium is a high-tech element, it has evoked interest in both biology and medicine. Germanium is not considered essential for life, nor does it present any threat to the environment (Enghag 2004). Notwithstanding the above, it is clear that the biological role of this element is still poorly examined. The most current research concerns organic germanium compounds and their effects. Various views are presented, from those that point to the advantages of taking germanium as a medicine (Asai 1981; Kamen 1987; Levine 1987; Faloona and Levine 1988; Goodman 1988a; Peng et al. 2000; Sellappa and Jeyaraman 2011), to those exposing the health risks resulting from the reckless use of germanium preparations (Schauss 1991a; Gerber and Leonard 1997; Tao and Bolger 1997).

The study of the biological role of inorganic germanium compounds like germanic acid (H_4_GeO_4_), which is the main germanium form in many natural waters, has not been given comparable attention. The element germanium is also not regulated or considered in drinking-water standards (WHO 2008).

In some East Asian countries such as Japan, the Republic of Korea, Taiwan, and China, germanium is valued and it is given a significant role in healthcare (Asai 1981; Kamen 1987; Satgé 2004), specifically: (1) appreciation for food rich in germanium, like shelf fungus, ginseng, sanzukon, waternut, boxthorn seed, garlic, and comfrey; (2) use of organic germanium dietary supplements; (3) production and consumption of synthetic waters heavily rich in germanium; (4) utilizing cosmetic products like pure germanium-containing skin massagers. Dr. Kazuhiko Asai, a Japanese chemist and germanium enthusiast, established in 1969 the Germanium Research Institute, the world’s first institute focused on studying germanium organic compounds. In Japan and the Republic of Korea, natural and synthetic waters rich in germanium are regarded as valuable for drinking and therapeutic purposes, and are available as bottled products such as Yaksan Water, Sansuryeo Water, Alpha Germanium Mineral Water, Germanium White Water, and Rota Germanium Mineral Water.

In Europe, non-high-tech interest in germanium, including the use of germanium-rich mineral water, is negligible when compared with East Asian countries as mentioned above. A few European bottled waters, like Dunaris (0.08 mg/L Ge; Bitschene and Sessler 2012) and Vincentka (0.11 mg/L Ge; Reimann and Birke 2010), are somewhat popular as beneficial for human health because of enrichment in germanium. Such perceptions are based on studies originally by Goldstein (1927, 1932), who successfully tested Dunaris water in the treatment of cancer and supposed that the germanium element is the active substance present in Dunaris water. Referring to the Goldstein’s and later works, Staufer (1985) attempted to arouse interest in germanium as a health-beneficial component of the curative waters. The geochemistry of curative and mineral waters are now much better recognized, and the subject of germanium health effects in balneology (medical hydrology, thermalism) should be pursued.

During our geochemical research on trace elements in curative waters used in health resorts located in the Polish part of the Sudetes Mountains (SW Poland), germanium was documented for the first time.

The aims of this publication are: (1) attempt to assess the origin of germanium in curative waters of the Sudetes Mountains, (2) indicate the geochemical/geological conditions conducive to finding mineral waters enriched in germanium in the Sudetes, (3) draw the attention of medical doctors interested in the therapeutic effects of drinking and bathing in medicinal springs to germanium as a possibly beneficial element in curative waters and worthy of further research.

## Germanium and human health

Germanium is naturally present in water and food, as it exists in soil, rocks, animals, and plants in organic and inorganic forms (Kang et al. 2001). Moreover, it is used in industry as a semiconductor and in the manufacture of phase-change optical magnetic discs, such as DVD-RAM and DVD-RW (Lin et al. 2006). The lack of recycling processes for these discs increases environmental contamination and the exposure to that metalloid (Kobayashi and Ogra 2009).

The biological function of germanium is not well known. It has been reported to inhibit a number of enzymes such as glutamic oxaloacetic transaminase, lactic dehydrogenase, alcohol dehydrogenase, and glutathione-S-transferase (Furst 1987). Its involvement in carbohydrate metabolism has been suggested, but not been proved until now (Goodman 1988b). Some literature has reported its beneficial effects on a number of conditions including cancer, HIV infection, liver disease, hypertension, arthritis, food allergies and malaria (Goodman 1988b; Furst 1987). However, germanium is not considered an essential nutrient. The studies (COT 2008) reported its very low population average intake at 0.1–1.5 µg/day and concluded that germanium dietary exposure was unlikely to be of toxicological concern.

Waters contain the inorganic form, mainly germanic acid (H_4_GeO_4_), while plant and animal-derived food contains organic compounds like *germanium 132* (*Ge*-*132*), a mixture of carboxyethyl germanium sesquioxide and 1-phenyl-2-carboxyethylgermanium sequisulfide; *sanumgerman*, a compound of lactate–citrate–germanium; and *spirogermanium*, a compound of 2-aza-8-german-spirodcane-2-propamine-8,8-diethyl-*N*,*N*-dimethylidichloride (Kang et al. 2001). Low levels (0.002–0.004 mg/kg) of the element are in cereal, bread, meat, and fish, while high levels (2–9 mg/kg) have been found in beans, tomato juice, oysters, tuna, garlic, aloe vera, and green tea (MAFF 1997). Germanium is not currently present in any market-approved medicines, although some clinical studies that included pharmaceutical preparations containing germanium compounds have been performed (Hirayama et al. 2003; Dhingra et al. 1986; Saiers et al. 1987).

Although germanium is considered to be a non-essential and non-harmful element, little still is known about its toxic effects and metabolism. Germanium is rapidly and extensively absorbed from the gastrointestinal tract after oral administration (Rosenfeld 1954; Furst 1987) and then fairly uniformly distributed between erythrocytes and plasma and transported to various organs and tissues in unbound form (Goodman 1988b; Rosenfeld 1954; Schauss 1991a). It is widely distributed in the body, while its accumulation and retention in the organs depend on the chemical form of germanium. Inorganic germanium tends to accumulate in the body, with the highest concentrations identified in the kidney, liver, spleen, gastrointestinal tract, and bones (Furst 1987). In the case of *Ge*-*132*, the highest concentrations have been found in the urinary bladder and lower concentrations in the digestive organs indicating a higher tendency to excrete the organic form of germanium than to accumulate it in organs (Schauss 1991a). Generally, germanium is excreted mainly in urine and only in low amounts via bile and faeces. Organic compounds are rapidly cleared and excreted more efficiently (Goodman 1988b). The biological half-life of germanium has been estimated at 1.5 days in case of whole body, 2 days in the liver, and 4.5 days in the kidneys (Furst 1987; Schauss 1991b). Therefore, the organ with the highest germanium accumulation is the kidneys. Germanium deficiency has not been identified or affirmed in animal studies, although it has been suggested to be a contributing factor in Kashin–Beck disease (KBD), an osteoarthritic condition affecting children in China and Russia (Peng et al. 2000). There are no reports concerning acute germanium toxicity, but prolonged consumption of inorganic germanium supplements has resulted in severe adverse effects including various organ dysfunctions and even death. Initial symptoms include anorexia, weight loss, fatigue, headaches, vomiting, diarrhoea, and muscle weakness, while longer intoxication causes renal dysfunction and failure accompanied by systemic symptoms such as muscle and nervous system damage (Nagata et al. 1985; Asaka et al. 1995; Schauss 1991b). Renal function does not return to normal even when germanium has been withdrawn (Van der Spoel et al. 1990). However, the toxicity of organic germanium compounds has been found to be lower and less severe.

The beneficial effects of germanium administration described in the literature, concern its use to prevent or treat cancer, HIV infection, autoimmune diseases, arthritis, and senile osteoporosis (e.g. Tanaka et al. 1984; Nagahama et al. 1986; Nakata et al. 1986; Aso et al. 1988; Goodman 1988b; Konno et al. 1990; Hirono et al. 1991; Fujii et al. 1993; Seaborn and Nielsen 1994; Hirayama et al. 2003). Phase I and phase II trials of spirogermanium as a therapy for cancer have been performed (Dhingra et al. 1986; Harvey et al. 1990). The molecular mechanism involved in beneficial applications of germanium could be explained by its preventive effect on the inhibition of gap junctional intercellular communication (Kang et al. 2001), which is an important event during the promotional stage of cancer. However, its negative effects could result from mitochondria-mediated oxidative stress and apoptosis (Lin et al. 2006). So far, the exact mechanisms are still not well known.

Germanium seems to be an interesting goal of research as it exerts prophylactic and therapeutic effects in the treatment of serious diseases. However, it tends to accumulate in organisms in a way that manifests in undesirable and even threatening side effects. Therefore, further studies should be performed to elucidate the exact molecular mechanism of germanium action, and to determine the safe dosage of germanium and duration of therapy.

## Germanium geochemistry and hydrogeochemistry

Germanium is widely distributed in the Earth’s crust and is mined primarily for use in the electronic and optical industries. The average germanium content in the upper continental crust is estimated at 1.4 ppm (Rudnick and Gao 2003). Germanium commonly demonstrates silicon-like geochemistry and is used as a tracer in petrogenetic processes leading to (re)crystallization of rocks in mantle, metamorphic, and volcanic–plutonic environments. Due to the substitution of Ge^4+^ for Si^4+^, most of germanium in the Earth’s crust is scattered in silicate minerals, which makes up as much as 90% of the mass of the Earth’s crust.

As a trace element, germanium rarely forms its own minerals, which are most often sulphides, like argyrodite Ag_8_GeS_6_, renierite (Cu,Zn)_11_(Ge,As)_2_Fe_4_S_16_, germanite Cu_3_(Ge,Ga,Fe)S_4_, briartite Cu_2_(Zn,Fe)GeS_4_. Germanium manifests an affinity not only to Si, but also to Zn, As, Fe, Cu, Sn, Ag, and accumulates mainly in sulphides (sphalerite ZnS, chalcopyrite CuFeS_2_, arsenopyrite FeAsS, pyrite FeS_2_), but rarely in iron oxy-hydroxides and silicates. Among oxides, the highest germanium concentrations are found in rutile, magnetite, and cassiterite, whereas among silicates, in topaz, epidote, garnet, and tourmaline (Ivanov 1996). Moreover, germanium also shows a preference to concentrate in organic matter, particularly in coal (e.g. Bernstein L. 1985; Höll et al. 2007). Germanium accumulates in trace and minor amounts in ore deposits, mainly in various types of Cu– Zn– Pb– Mo– Au- sulphide ore deposits. Sphalerite is the most important of all germanium-containing minerals. Germanium is mostly recovered from sphalerite ores and from lignite and coals (Frenzel et al. 2014).

In fresh groundwater (of total dissolved solids below 1 g/L), germanium belongs to trace components, i.e. substances which occur usually at concentrations below 0.1 mg/L. An increased germanium level is mainly associated with thermal waters, waters with either very low or very high pH, and saline waters (Rosenberg 2009). For example, germanium was found in increased amount in CO_2_-rich thermal waters, methane-containing waters, nitrogen-rich waters, acid thermal water in the oxidation zone of sulphide deposits, and alkaline sodium-dominated thermal waters (Ivanov 1996).

In thermal waters, germanium concentration ranges widely, from undetectable to almost 300 µg/L, but rarely exceeds 50 µg/L (Table 1). Increased germanium content is usually found in alkaline and/or thermal groundwater, especially in active volcanic zones and/or in bedrock built of reactive silicate minerals, as in young volcanic rocks. However, the reaction of thermal alkali-rich waters with organic-rich sedimentary rocks might also favour very high germanium concentrations (Bernstein 1985).

**Table 1:** Concentration of germanium in various groundwater, including bottled waters

Water type, location, water sample sizes	Range (µg/L)	Median (µg/L)	Data source
Thermal water, Vichy, France (*N* = 1)	25	–	Bardet (1914)
Thermal water, Senami, Japan (*N* = 1)	30	–	Kuroda (1939)
Thermal water, Beppu, Japan (*N* = 24)	< 2–150	–	Kawakami et al. (1956)
Thermal spring waters, USA and Iceland (*N* = 7)	< 10–40	–	El Wardani (1957)
Thermal waters, Hokkaido and Honshu islands, Japan (*N* = 84)	0.4–43.3	7.8	Uzumasa et al. (1959)
Thermal waters, New Zealand (*N* = 38)	1–128	52.5	Koga (1967)
Carbonate thermal waters, Pamir and Caucasus mountains (*N* = 16)	12–140	32.5	Kraynov (1967)
Groundwaters of ore deposits, USSR (*N* = 26)	0.5–48	3.0	Goleva and Vorobjeva (1967)
Mineral (Na-HCO_3_-Cl) waters and Na-Cl saline waters, oil deposits, USSR (*N* = 36)	0.3–8.5	3.25	Nuriev et al. (1968)
Thermal waters, Vosges, France (*N* = 8)	< 0.5–15.4	–	Gijbels et al. (1983)
Thermal waters, Iceland (*N* = 132)	0.5–52.5	6.1	Arnórsson (1984)
Thermal waters, Vals Les Bains and Vichy, Massif Central, France (*N* = 35)	0.5–47.9	13.1	Criaud and Fouillac (1986)
Mineral waters, deep gold mines, South Africa (*N* = 12)	< 0.05–276	–	Duane et al. (1997)
Thermal spring waters, Baikal area (*N* = 4)	0.98–9.81	–	Kenison Falkner et al. (1997)
Groundwater (fresh) in crystalline bedrocks, Norway (*N* = 476)	< 0.002–1.5	0.017	Frengstad et al. (2000)
Thermal waters, Iceland (*N* = 88)	0.001–23.6	2.66	Elmi (2009)
European bottled waters (only waters with Ge concentration ≥ DL) (*N* = 882)	0.03–110	0.09	Reimann and Birke (2010)
Thermal spring waters, Lesvos Island, Greece (*N* = 6)	< DL–13	–	Tziritis and Kelepertzis (2011)
Fresh and mineral groundwaters, Bieszczady Mountains, Poland (*N* = 23)	0.08–35.8	7.5	Dobrzyński et al. (2011)
CO_2_-rich and thermal curative waters, the Sudetes Mountains, Poland (*N* = 33)	0.025–10.62	1.01	This study

The most important species of germanium in aqueous solutions are germanic acid (H_4_GeO_4_) and Ge–fluoride complexes at high fluoride concentrations (Wood and Samson 2006). Methylgermanium species (monomethylgermanium and dimethylgermanium) have been found in surface waters (Lewis et al. 1988). High solubility of tetramethylgermanium was proposed as responsible for high germanium concentrations in mineral waters occurring in carbonaceous sedimentary rocks (Ivanov 1996).

The most important factors affecting germanium concentrations appear to be temperature and geochemistry and mineralogy of reservoir rocks. Generally, germanium concentrations in groundwater are controlled by the dynamic equilibrium between oppositely acting processes, i.e. release from source solid phases into a solution and the immobilization of the element by precipitation and adsorption. The decay of mineral source phases and increase in Ge concentration are supported by the raising of temperature and pH increase, which favours germanic acid dissociation. Immobilizing the element into solid sink phases depends on changes of solution chemistry and temperature, e.g. during the gradual decrease in temperature caused by thermal groundwater ascending towards the surface or mixing with shallower groundwater of contrasting composition or temperature.

## Research scope: methods

**Fig. 1:**
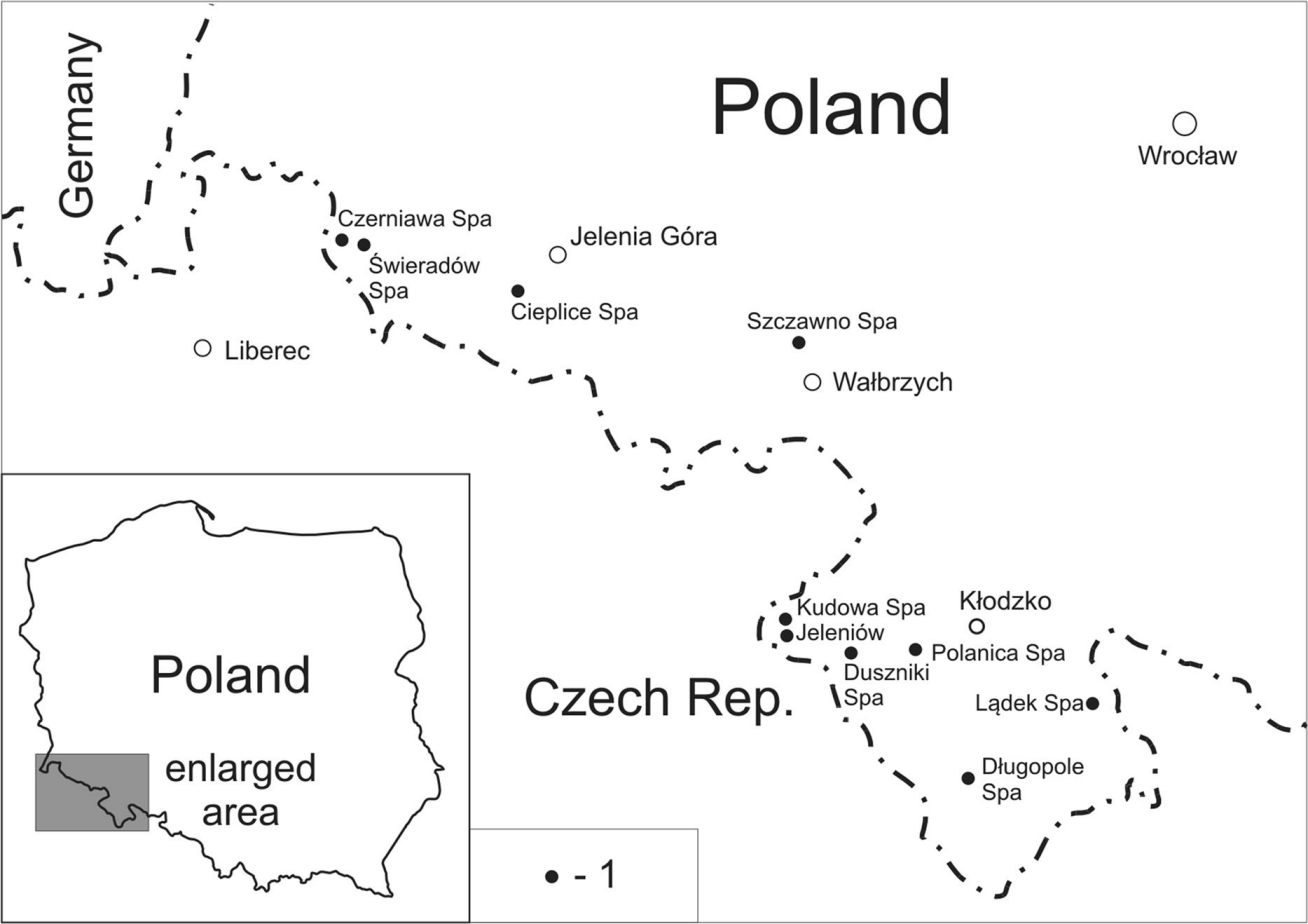
Location of sites where curative waters were surveyed (closed circles; towns are open circles)

The chemical composition of curative and mineral waters from 33 water intakes located at health resorts in the Sudetes Mountains (Poland) (Fig. 1) was studied. Mineral CO_2_-rich water from Jeleniów, which is not used for balneotherapy (treatment of disease by bathing in, inhalation, or drinking mineral waters), was also examined because of its similarities to the curative waters of Kudowa Spa. For the first time, germanium was analysed in curative waters of the Sudetes.

Study of the water composition included field measurements of physico-chemical parameters and chemical analyses. Redox potential and pH were measured in the field in flow cell (Eijkelkamp) with a PW9424 meter (Philips) accompanied with temperature probe PW9516/08 ATC, combined electrode CE50 and Pt–Ag/AgCl redox electrode (Corning). Specific electric conductivity (SEC) was measured by L21 conductometer (Eijkelkamp). Water samples were filtered in the field by cellulose nitrate membrane filters (Sartorius) of 0.45-µm pore size, preserved by ultra-pure HNO_3_ (Merck), and stored in LDPE containers (Nalgene). Bicarbonates and chlorides were analysed volumetrically, and sulphates, spectrophotometrically. Other components were determined by ICP-MS method (ACME, Canada). Geochemical speciation modelling of water was performed by using the PHREEQC programme, ver. 3.3 (Parkhurst and Appelo 2013) with the LLNL thermodynamic database. Data for parameters used in the discussion presented in the paper are collated in Table 2.

**Table 2:** Selected physico-chemical parameters of studied curative waters

Intake number	Intake, locality	SEC^a^	Ionic strength^b^	*T*	pH	pe	Si	Ge	Fe	As
		µS/cm	mmol/L	°C			mg/L	µg/L	mg/L	µg/L
1	Zdzisław (Lądek)	237	3.035	44.3	9.22	− 2.522	25.01	3.52	0.04	0.7
2	Chrobry (Lądek)	226	2.855	27.5	8.94	− 1.939	25.38	3.47	0.09	1.3
3	Wojciech (Lądek)	227	2.608	29.8	8.95	− 1.281	20.69	2.74	0.01	1.1
4	Skłodowska-Curie (Lądek)	220	2.069	26.0	8.90	− 0.172	14.38	1.86	0.10	0.8
5	Dąbrówka (Lądek)	221	2.637	20.0	8.96	− 1.310	23.47	3.16	0.23	1.4
6	Jerzy (Lądek)	209	2.350	28.0	9.33	− 1.777	16.93	2.20	0.01	2.0
7	Sobieski (Cieplice)	1001	14.289	21.0	6.63	5.873	23.13	3.71	0.28	23.6
8	C-2 (Cieplice)	777	9.923	58.8	8.35	− 2.133	44.28	6.29	0.03	52.0
9	Nowe (Cieplice)	751	9.835	29.4	7.59	4.487	38.26	4.97	0.03	47.1
10	Marysieńka (Cieplice)	776	9.676	21.6	7.98	5.149	41.30	5.31	0.12	50.7
11	J-150 (Jeleniów)	1558	19.137	12.5	5.68	4.216	27.45	3.54	8.09	1099.1
12	Moniuszko (Kudowa)	3285	35.392	16.1	6.14	2.380	7.01	3.94	6.86	1375.7
13	Marchlewski Nowy (Kudowa)	1780	15.392	13.4	5.83	5.119	6.15	2.03	2.65	337.3
14	K-200 (Kudowa)	3165	47.610	13.3	6.30	3.346	48.48	10.62	13.21	3532.7
15	Jan Kazimierz (Duszniki)	1529	19.268	16.7	6.10	4.164	18.97	0.88	6.47	145.6
16	Pieniawa Chopina (Duszniki)	2015	15.461	17.9	6.16	3.762	14.36	0.43	6.50	87.2
17	B-39 (Duszniki)	1816	15.762	18.4	6.17	4.024	16.28	0.67	5.73	132.3
18	B-4 (Duszniki)	2525	37.177	17.2	6.30	2.994	31.93	1.16	15.37	219.3
19	Wielka Pieniawa (Polanica)	1425	15.669	12.5	5.65	4.410	6.77	0.41	4.78	86.6
20	Józef 2 (Polanica)	762	8.664	11.8	5.82	4.946	4.79	0.13	2.30	10.6
21	P-300 (Polanica)	2455	40.181	15.5	6.30	2.498	6.41	1.01	9.05	104.1
22	Marta (Szczawno)	2175	27.091	12.9	5.81	5.199	15.24	0.59	4.14	0.025
23	Młynarz (Szczawno)	1952	25.451	14.9	5.95	5.540	12.68	0.30	2.49	0.025
24	Dąbrówka (Szczawno)	2125	24.317	13.9	5.84	5.525	15.00	0.40	3.69	0.6
25	Mieszko (Szczawno)	3430	39.415	13.1	6.03	5.411	16.87	0.68	4.98	0.025
26	Renata (Długopole)	1261	14.601	11.4	5.57	5.279	23.78	0.34	14.28	2.8
27	Kazimierz (Długopole)	1051	13.884	11.0	5.52	5.345	24.41	0.30	13.79	2.4
28	Emilia (Długopole)	937	13.012	10.9	5.54	5.420	22.57	0.24	14.09	5.9
29	4 (Czerniawa)	2365	36.202	11.6	5.79	3.414	37.71	0.87	19.78	0.6
30	Górne (Świeradów)	344	3.983	11.5	5.03	5.966	16.55	0.14	4.36	0.6
31	1A (Świeradów)	1129	14.669	9.7	5.40	4.573	35.42	0.76	35.33	2.4
32	2P (Świeradów)	1760	23.453	9.3	5.56	3.943	34.74	4.33	290.38	0.025
33	MSC^c^ (Świeradów)	77.8	1.051	8.8	5.64	4.062	9.12	0.025	0.20	1.1

Intake number	Intake, locality	Zn	Ca	Mg	Na	K	HCO_3_	SO_4_	Cl
		µg/L	mg/L	mg/L	mg/L	mg/L	mg/L	mg/L	mg/L
1	Zdzisław (Lądek)	111.7	6.37	0.24	53.09	0.86	60.6	15.2	8.9
2	Chrobry (Lądek)	7.8	5.38	0.13	56.53	0.91	60.8	15.8	7.1
3	Wojciech (Lądek)	0.4	4.96	0.26	45.25	0.78	53.1	21.4	7.1
4	Skłodowska-Curie (Lądek)	0.5	2.75	0.13	29.87	0.52	53.1	19.0	7.1
5	Dąbrówka (Lądek)	19.0	5.58	0.24	48.71	0.89	53.1	18.2	7.1
6	Jerzy (Lądek)	0.5	4.48	0.26	36.74	0.56	53.1	15.0	7.1
7	Sobieski (Cieplice)	40.3	95.45	12.44	117.26	46.51	294.7	174.5	64.9
8	C-2 (Cieplice)	1.4	10.16	0.06	188.64	5.44	162.3	149.0	43.6
9	Nowe (Cieplice)	15.8	18.85	1.10	169.32	7.07	172.4	149.8	41.8
10	Marysieńka (Cieplice)	38.2	9.79	0.12	185.51	5.66	158.5	147.7	41.1
11	J-150 (Jeleniów)	2.0	142.78	45.90	228.85	38.28	874.0	89.1	30.1
12	Moniuszko (Kudowa)	13.2	272.16	42.73	377.17	49.66	2276.0	196.6	78.0
13	Marchlewski Nowy (Kudowa)	24.3	110.69	20.31	138.80	18.53	1287.5	143.6	49.6
14	K-200 (Kudowa)	14.2	298.90	112.53	591.48	86.33	2111.2	202.4	72.7
15	Jan Kazimierz (Duszniki)	13.8	170.56	42.60	129.48	74.46	1073.9	44.0	8.9
16	Pieniawa Chopina (Duszniki)	21.6	111.77	29.52	72.46	42.86	1507.1	52.7	8.9
17	B-39 (Duszniki)	16.9	122.62	33.55	73.30	46.99	1305.8	47.1	10.6
18	B-4 (Duszniki)	29.4	323.79	91.02	240.97	138.81	1866.0	53.9	11.5
19	Wielka Pieniawa (Polanica)	0.1	205.74	23.89	60.94	34.56	1043.4	25.9	7.1
20	Józef 2 (Polanica)	34.1	109.08	13.25	25.04	19.13	488.1	24.4	10.6
21	P-300 (Polanica)	0.5	502.43	61.23	147.44	59.83	1983.1	29.5	7.1
22	Marta (Szczawno)	12.0	122.70	69.91	477.84	15.06	1633.0	139.1	26.0
23	Młynarz (Szczawno)	2.1	111.36	73.65	372.01	23.75	1361.0	147.3	66.0
24	Dąbrówka (Szczawno)	2.2	120.01	57.66	439.89	11.29	1270.0	104.1	29.5
25	Mieszko (Szczawno)	160.1	143.79	92.21	761.53	24.06	1845.0	226.3	73.0
26	Renata (Długopole)	2.6	130.58	57.97	74.81	9.26	868.0	19.0	10.3
27	Kazimierz (Długopole)	5.9	127.41	54.43	67.66	8.79	558.3	34.0	12.1
28	Emilia (Długopole)	21.7	122.56	49.13	59.72	8.01	551.0	35.0	10.3
29	4 (Czerniawa)	6.0	347.08	158.11	125.39	14.36	1924.1	4.0	8.9
30	Górne (Świeradów)	16.1	34.27	14.26	12.47	6.02	153.9	20.2	9.8
31	1A (Świeradów)	38.8	115.16	74.60	40.27	17.58	447.0	8.0	7.1
32	2P (Świeradów)	19.4	128.13	84.81	60.03	24.47	1992.0	6.0	30.1
33	MSC (Świeradów)	7.4	6.46	2.49	5.32	1.03	18.1	14.5	4.4

^a^Specific electric conductivity

^b^Calculated by the PHREEQC program (Parkhurst and Appelo 2013)

^c^*MSC* Maria Skłodowska-Curie intake

Due to the significant differences, hydrochemical data were interpreted by PCA analysis, separately for cold CO_2_-rich waters and thermal waters. Particular numerical series were subjected to the analysis of statistical distribution (by means of the W Shapiro–Wilk test) before determination of substitute variables. Asymmetric and multimodal data were mathematically transformed aimed at the change of the distribution into a more normal one, in accordance with the recommendations by Norcliffe (1986). Next, the data for calculations were standardized. Substitute variables were determined from the prepared data matrix as the primary components. The matrix was rotated by the Varimax normalized method in order to maximize variance in the data columns. The primary components, important for the explanation of the variance of the data matrix, were determined by the self-organizing method. Statistical calculations were performed in the STATISTICA (ver. 7.1) software.

The chemical composition of groundwater was interpreted from the viewpoint of available data on aquifer geochemistry and mineralogy to indicate the relationship between germanium and co-occurring elements. Discussion of germanium origin in the waters studied focuses on key physico-chemical parameters and chemical elements which presumably affect Ge hydrogeochemistry.

The content of germanium, silicon, arsenic, iron, and zinc in studied groundwater was compared with the weighted mean composition of the Earth’s upper crust (after data by Rudnick and Gao 2003) with the aim of deciphering the relationships between the elements. Germanium-to-(Si, As, Fe, Zn) mass ratios in groundwater have been normalized by dividing the ratios in groundwater by the relevant mean ratios for upper crust rocks. This proposed complex parameter takes into account element contents in bedrock and also illustrates the scale of relative enrichment/reduction in groundwater chemistry with respect to the Earth’s crust composition.

## Germanium geochemistry in curative waters. Results and discussion

Curative waters are used for balneotherapy in nine spas in the Sudetes (Fig. 1). A significant proportion of the geological settings of the region are magmatic and metamorphic rocks, which is reflected in the chemistry of both fresh and mineral waters. Despite the diversity of water chemistry caused by local lithologies and hydrogeological conditions, generally, two primary types of curative waters can be identified in the Sudetes: (1) CO_2_-rich (acidulous) cold (< 20 °C) waters and (2) thermal waters. The CO_2_-rich waters are of bicarbonate type with various (dominated by Ca, Mg, Na) cationic composition and also with high content of Fe. Thermal waters are dominated by sulphates, bicarbonates, and sodium and are usually rich in fluoride and contain increased H_2_S, and/or Rn, and/or silicic acid.

**Fig. 2:**
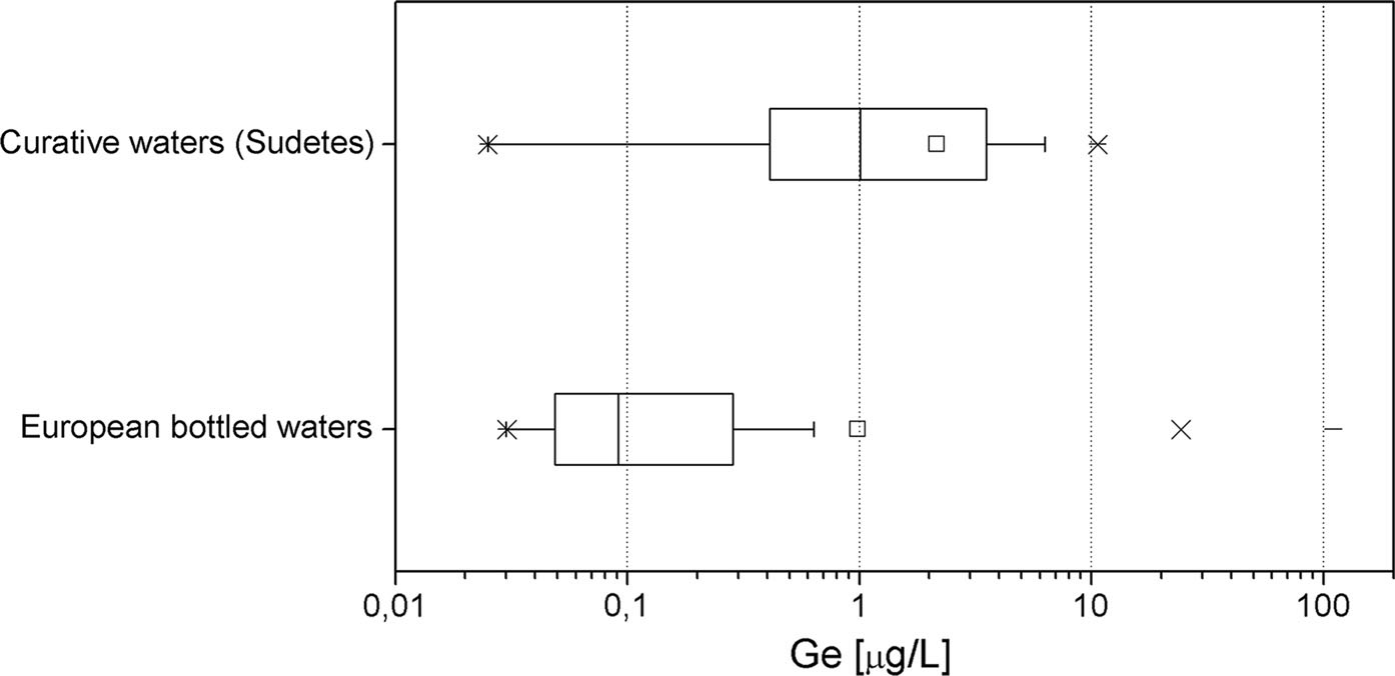
Box charts for germanium concentration in studied curative waters from the Sudetes (*N* = 33) and European bottled waters (*N* = 882; data after Reimann and Birke 2010). Only European bottled waters in which germanium was detected (i.e. ≥ 0.03 µg/L) were included

Germanium concentrations in curative waters studied vary between 0.025 and 10.62 µg/L and are lower than in most thermal waters from other geo-environments (Table 1). Curative waters from the Sudetes present a median germanium concentration of one order of magnitude higher compared to European bottled waters (Fig. 2).

Germanium scattering in silicate minerals is commonly found in many geological environments because it correlates with silicon and consequently is found in relation to Si in groundwater. However, an unusual property of germanium is that it manifests an affinity for siderophile, lithophile, chalcophile, and biolithophile compounds or phases. Germanium might be enriched, e.g. in sulphide minerals (mainly Zn-, Cu-, Fe- and As-sulphides), oxidized zones of germanium-bearing sulphides, iron oxides, late- and post-magmatic rocks (pegmatites, greisens, and skarns), coals, and lignites, which significantly complicates elucidating the origin of germanium in groundwater.

Statistical PCA analysis helped to clarify the relationships between parameters and to explain the majority of observed geochemical variations. In CO_2_-rich waters, three significant substitute variables were determined (Table 3) which explains 76% of the total variance and take into account nine of twelve analysed hydrochemical parameters (variables). In PC1, sulphates, chlorides, sodium, temperature, and pH were distinguished. This manifests a positive relation between solutes (SO_4_, Cl, Na) typical for a deep water component, and temperature and pH. PC2 includes silicon, germanium, and iron which implies the role of iron-bearing silicates, possibly accompanied by oxide/hydroxides, in release germanium into and control this element in solutions. An inverse relation between arsenic and redox potential (in substitute variable PC3) confirms the decrease in the mobility of arsenic in water due to oxidation of As(III) to As(V), and immobilization of arsenic acid anions in more oxidative conditions, probably due to adsorption onto iron oxy-hydroxides.

**Table 3:** Factor loads of hydrochemical data matrix for CO_2_-rich waters

	PC1	PC2	PC3
Eigenvalue	5.08	2.41	1.60
Variance explained (%)	42.3	20.1	13.6
Temperature	**0.734**	− 0.152	− 0.442
H^+^ (logarithm)^a^	− **0.785**	− 0.037	0.462
Redox potential	− 0.188	− 0.347	**0.805**
Si	− 0.114	**0.844**	0.090
Ge (logarithm)	0.519	**0.709**	− 0.219
Fe (logarithm)	− 0.061	**0.915**	0.0001
As (logarithm)	0.275	− 0.0003	− **0.756**
Zn (cube root)	0.248	0.145	0.488
K (logarithm)	0.680	0.418	− 0.439
Ca + Mg + HCO_3_	0.580	0.589	− 0.297
SO_4_ (logarithm)	**0.866**	− 0.283	0.168
Na + Cl (logarithm)	**0.865**	0.308	0.170

Crucial values (> |0.7|) are in bold

^a^The mathematic formula used to normalize statistical distribution

In thermal waters, three substitute variables were also determined (Table 4) which explains up to 90% of variance and contains all of the analysed hydrochemical parameters. PC1 covers 8 of 12 analysed variables, Ge, Si, and pH in opposition with Na + Cl, K, Ca + Mg + HCO_3_, As, and SO_4_. Large numbers of variables in PC1 likely results from the small set size. This substitute variable, with mostly high factor loadings, might be explained as the effect of bedrock decay boosted by temperature rise. This process is likely responsible for germanium and silicon co-release from silicate minerals. It is interesting that in the case of thermal waters, pH seems to have a bigger influence on arsenic concentration than redox potential. Increase in arsenic concentration in more alkaline thermal waters might be caused by increase in competing OH^−^ groups activity while simultaneously decreasing preference for anion adsorption resulting from pH increase. The substitute variable PC2 includes temperature in opposition with redox potential and involves the increase in reductive conditions with the increase in temperature. PC3 includes iron and zinc and might suggest that both elements are derived from the same source, most likely sulphide minerals.

**Table 4:** Factor loads of hydrochemical data matrix for thermal waters

	PC1	PC2	PC3
Eigenvalue	7.15	2.43	1.29
Variance explained (%)	59.6	20.3	10.7
Temperature (logarithm)	0.281	**0.860**	− 0.248
H^+^ (logarithm)	**0.703**	− 0.588	0.220
Redox potential (logarithm)	0.395	− **0.875**	0.072
Si	**0.906**	0.246	0.105
Ge	**0.936**	0.251	0.152
Fe (logarithm)	0.036	− 0.466	**0.762**
As (reciprocal)	− **0.847**	0.344	0.106
Zn (logarithm)	0.291	− 0.008	**0.913**
K (reciprocal)	− **0.933**	0.147	− 0.298
Ca + Mg + HCO_3_ (reciprocal)	− **0.910**	0.243	− 0.228
SO_4_ (root of − 3°)	− **0.817**	0.423	0.088
Na + Cl (reciprocal)	− **0.954**	0.036	− 0.268

Crucial values (> |0.7|) are in bold

**Fig. 3:**
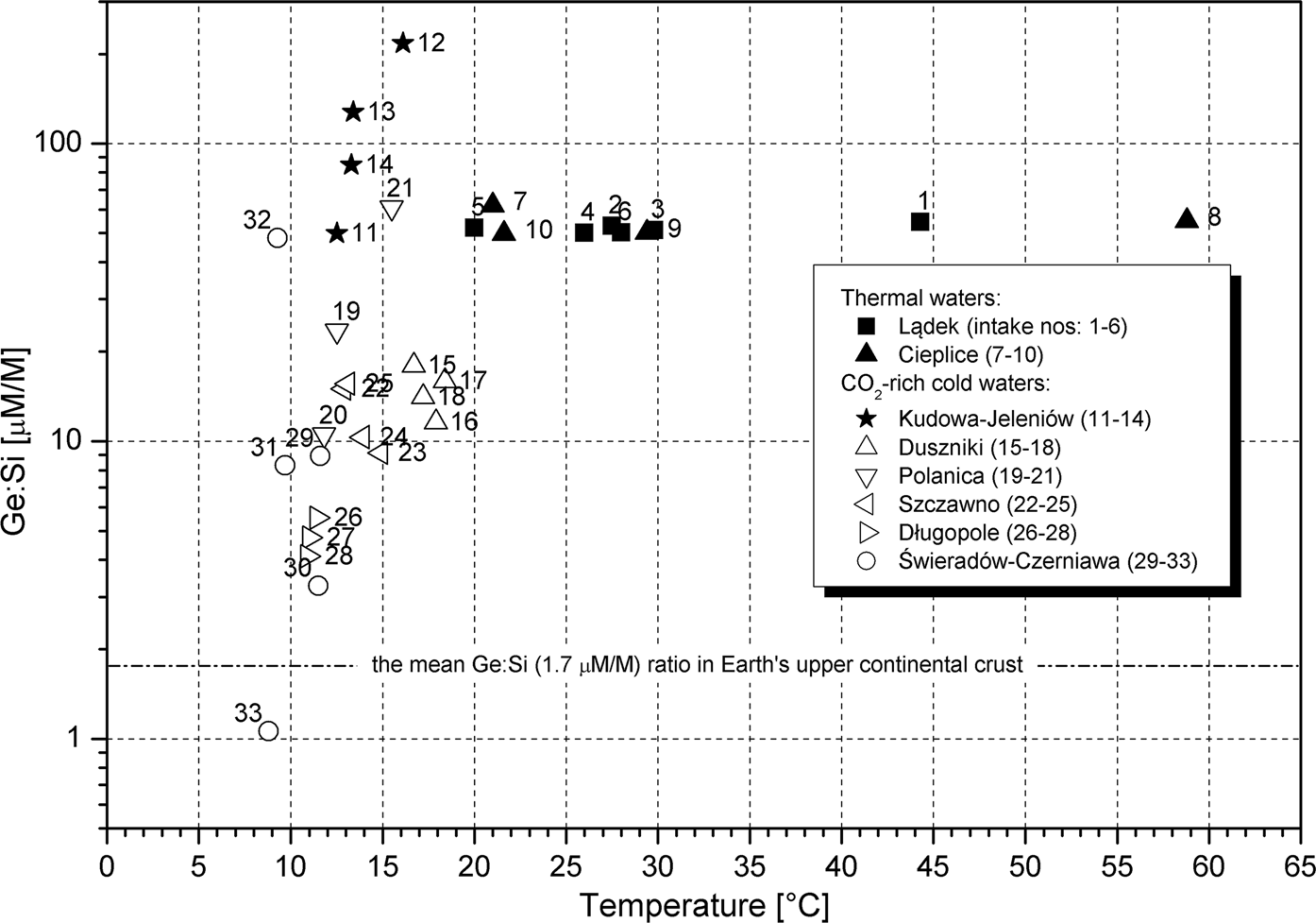
Ge/Si [µM/M] ratio versus temperature of groundwater. Intake numbers as in Table 2

The studied curative waters (Table 2), except for one (sample no. 33), are enriched in germanium with respect to silicon when compared to the composition of the upper continental crust (Fig. 3). Sample no. 33 originated from an unconfined aquifer in the weathering cover and represents water with the lowest mineralization among the studied waters (Table 2). The Ge–Si [µM/M] ratio, a measure of enrichment in germanium, varies between 1.06 and 217.25 and reveals a clear distinction between CO_2_-rich waters and thermal waters (Fig. 3). The first group shows a sharp increase in the Ge–Si ratio with increasing temperature. The quasi-constant Ge–Si [µM/M] ratios (between 49.7 and 62.0, mean 52.8) in thermal waters (Lądek, Cieplice) indicate that chemical characteristics likely acquired in the deep part of hydrothermal systems are still controlled during conductive cooling or mixing with low-temperature shallow groundwater as thermal waters ascend to the surface. Both Cieplice and Lądek thermal waters show similar Ge–Si ratios, denoting process(es) independent of hydrogeological conditions. The thermal waters of Cieplice and Lądek occur in mineralogically similar aquifer rocks (Cieplice waters in granitoids of the Karkonosze massif; Lądek waters in granite gneisses).

Evans and Derry (2002) documented the role of vein quartz in controlling the Ge–Si relationship in Himalayan thermal (*T* = 20–70 °C) waters. The neo-formed silica in Cieplice and Lądek geothermal systems are likely solid phases which control germanium (and silicon) concentrations in both thermal waters. Geochemical modelling of thermal (21–78 °C) waters from Cieplice, Karpniki, and Staniszów sites in the Jelenia Góra geothermal system (Sudetes, Poland) suggested that a secondary silica form, possibly quartz, takes part in incongruent transformation of rock-forming silicates and play a significant role in silicon and germanium control in mentioned thermal waters (Dobrzyński et al. 2017).

**Fig. 4:**
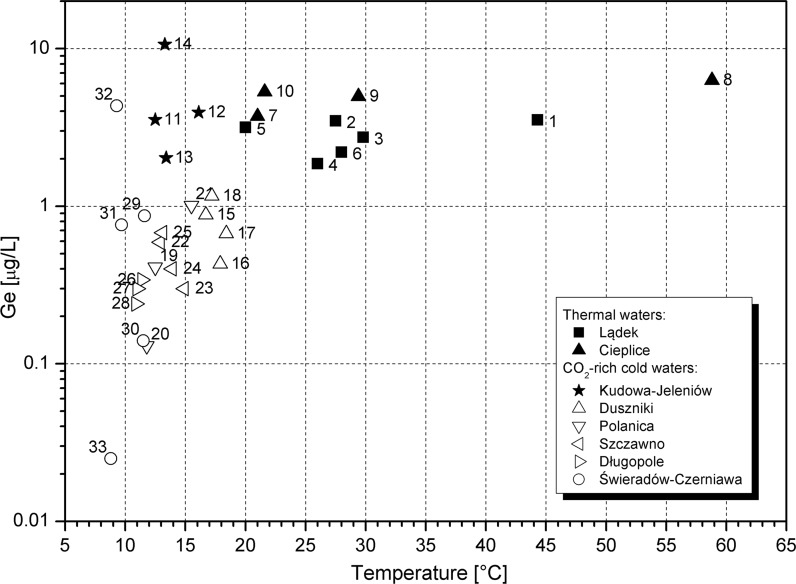
Germanium concentration versus temperature of groundwater. Intake numbers as in Table 2

The lowest germanium concentration was found in groundwater which has the lowest temperature (8.8 °C in water from the Maria Skłodowska-Curie intake (no. 33) in Świeradów health resort; Table 2). An anticipated increase in concentrations of germanium with the increase in water temperature is not common (Fig. 4) in studied waters. A germanium concentration of 3–10 µg/L can be found through the whole range of water temperatures studied, i.e. between 9 and 59 °C. This might suggest a preponderant influence of aquifer mineralogy and geochemistry, in particular, the role of reactive germanium-containing minerals. In the case of most CO_2_ waters, no apparent relationship between germanium and temperature is found. The high variability of germanium concentrations (0.03–10.62 µg/L) appears at small temperature fluctuations (8.8–18.4 °C). In contrast, germanium in thermal waters remains quasi-constant (Fig. 4) despite high variations in temperature (20–59 °C).

**Fig. 5:**
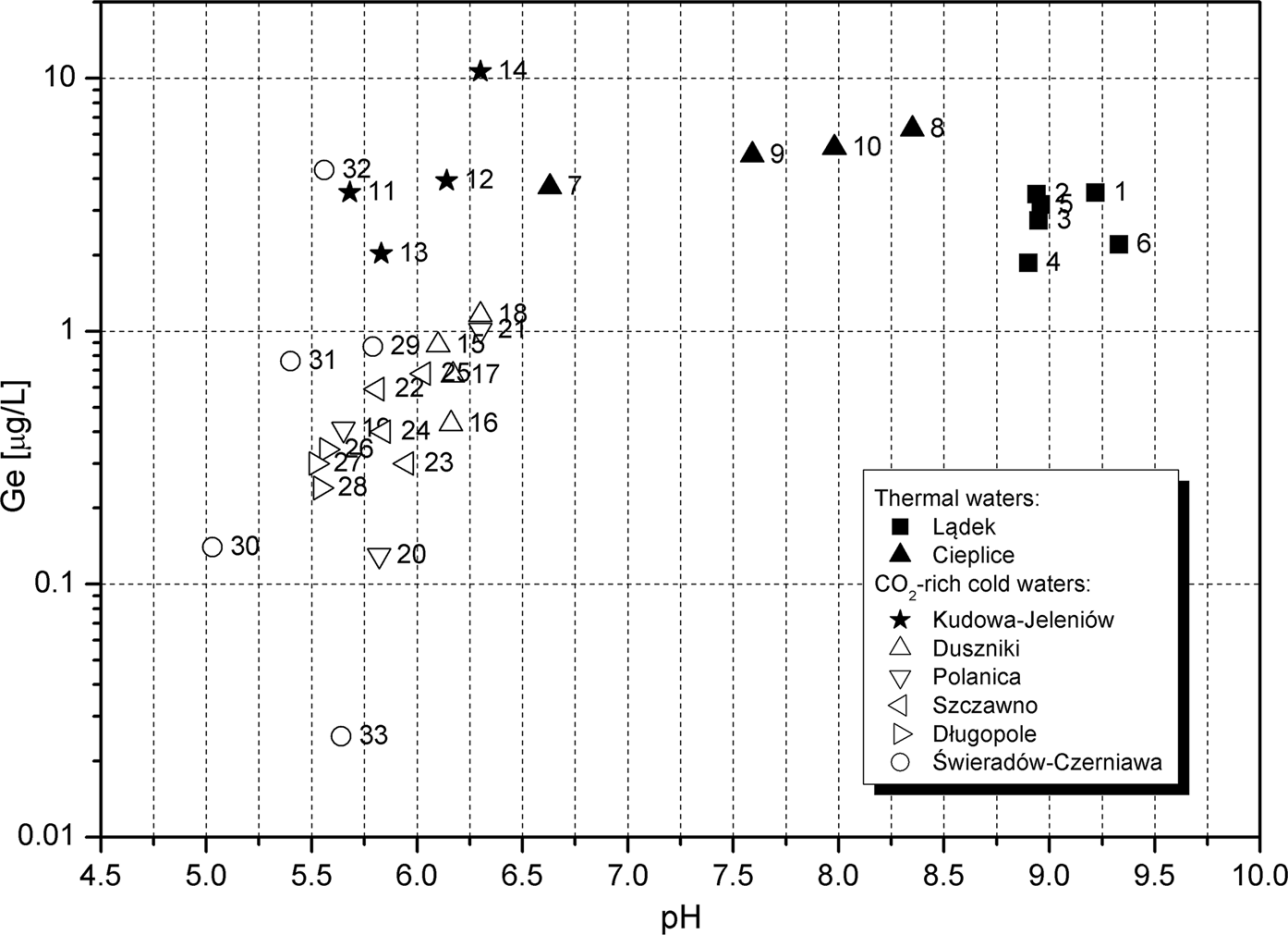
Germanium concentration versus pH of groundwater. Intake numbers as in Table 2

The solubility of germanium, analogous to silicon, might be expected to be pH dependent with the highest concentrations in alkaline waters (as a co-effect of dissociation of germanic and silicic acids and an increase in the solubility of silicate minerals) and extremely acidic waters (due to the decomposition of germanium-bearing phases in an aggressive environment). The Ge-pH pattern (Fig. 5) is generally similar to that for the Ge-temperature pattern (Fig. 4). An increase in germanium concentration with increasing pH is barely seen in studied waters (Table 2). As a result, Ge concentrations of 2–6 µg/L can be found both in the CO_2_-rich water of Kudowa (at pH=5.7–6.3) and in the thermal waters of Cieplice (pH=6.6–8.4) and Lądek (pH=8.9–9.3).

In all studied groundwater systems, germanium solute positively correlates with the ionic strength of a solution (Online Resource 1). The increase in ionic strength of groundwater at particular hydrogeological systems is caused by a longer groundwater transit time (i.e. longer water–rock contact time), which in turn ultimately promotes an incremental increase in germanium concentrations. Differences of the Ge-ionic strength patterns are fingerprints of aquifer lithology, reactivity of minerals, and hydrogeological conditions.

The effect of water residence time in the hydrogeological system and the reactivity of the aquifer rock appear to be easily noticeable in CO_2_-rich waters. Dissolved (bi)carbonates which strongly depend on CO_2_ influx and hydrolytic decay of minerals, correlate with germanium concentration (Online Resource 2). A gradual increase in germanium concentration seems to be feasible at further development of water–rock interactions, even after reaching the apparent limit of bicarbonate concentration (at about 2000 mg/L of HCO_3_).

**Fig. 6:**
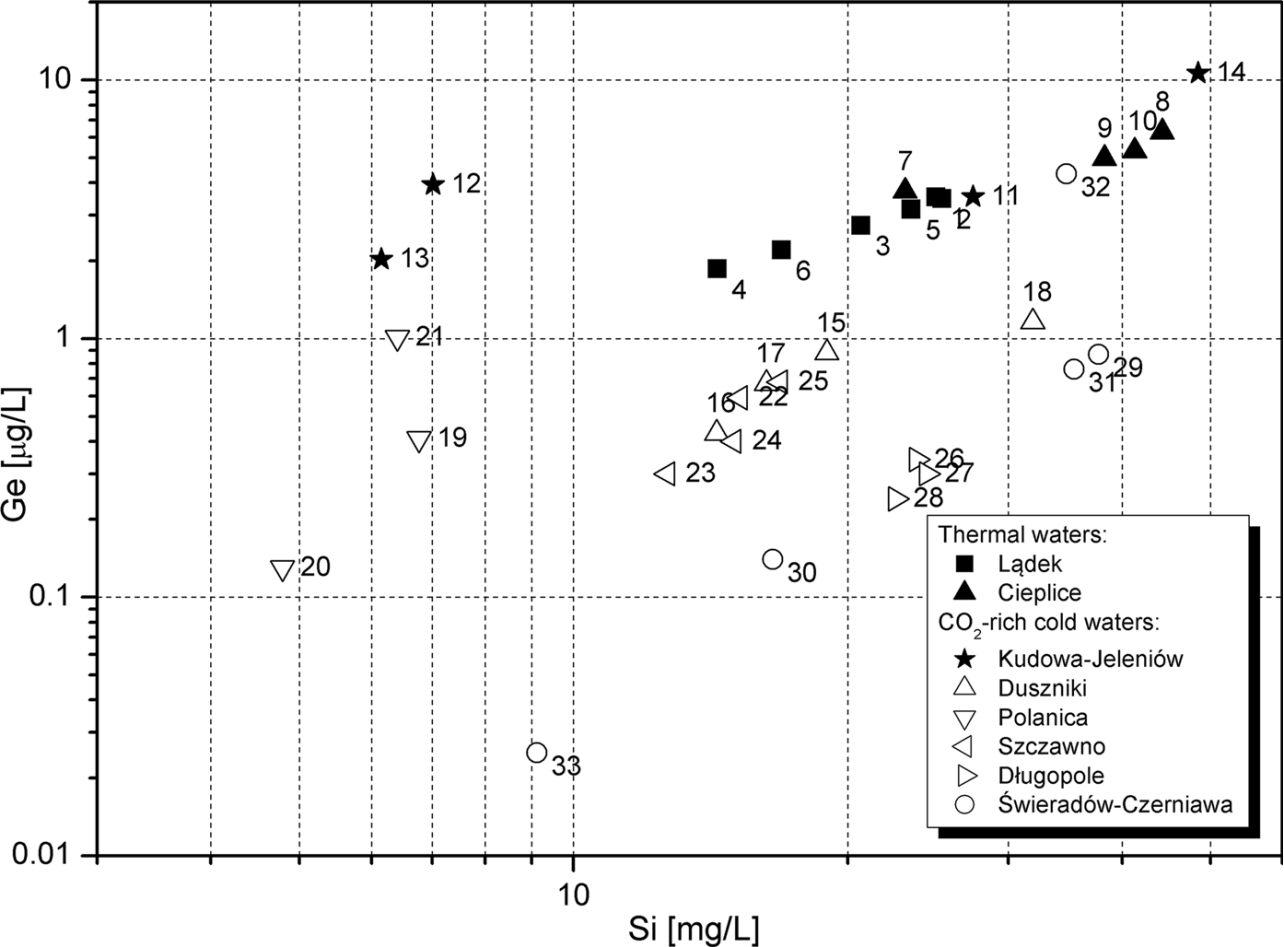
Germanium versus silicon in groundwater. Intake numbers as in Table 2

The effect of the silicon-like geochemistry of germanium is clearly visible in studied groundwaters. The decomposition and/or transformation of primary/secondary silicate minerals is very likely to be responsible for releasing germanium into solution and its positive correlation with dissolved silicon (Fig. 6). The chemical character of waters from different hydrogeological systems is likely caused by the mineralogical composition of aquifer rocks, while hydrochemical diversity within a particular system results from local hydrogeological conditions in the alimentation zones of individual water intakes.

Germanium has an affinity to iron, especially in Fe sulphides and oxy/hydroxides. Chemistry of the studied waters reveals a large variability in terms of Ge–Fe pattern (Online Resource 3). The alkaline thermal waters of Cieplice-Lądek, with a quasi-constant germanium concentration of 2–6 µg/L, contain less iron than CO_2_-rich slightly acidic (pH 5.0–6.3) waters. A positive correlation between Fe and Ge elements found in the waters of Świeradów-Czerniawa, Szczawno, Kudowa-Jeleniów, Polanica (Online Resource 3) suggests an important role of the iron-bearing phases by germanium release due to the breakdown of silicates and sulphides, and/or by germanium control by secondary Fe oxide/hydroxides. This leads to a different conclusion from the opinion of Kurtz et al. (2002) who, based on weathering zone studies, emphasized the dominant role of secondary aluminosilicates in controlling the behaviour of germanium.

**Fig. 7:**
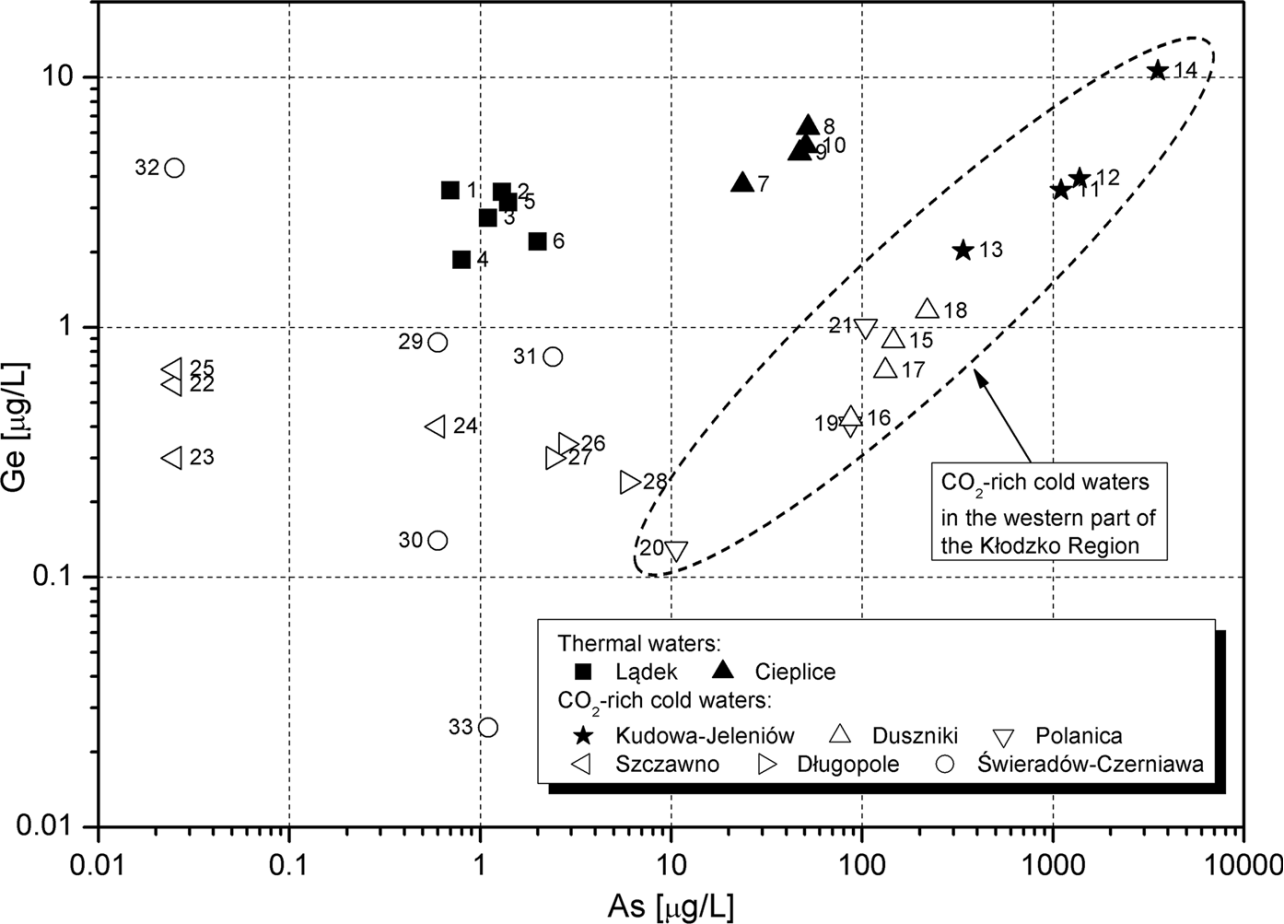
Germanium versus arsenic in groundwater. Intake numbers as in Table 2

The Ge–Zn pattern (Online Resource 4) does not show a relationship between the elements, while the Ge–As signature markedly distinguishes groundwater systems (Fig. 7). A pronounced positive relationship between concentrations of germanium and arsenic in waters is seen only in the SE part of the Intra-Sudetic Basin, in waters from Kudowa, Jeleniów, Duszniki, Polanica (KJDP) sites, which are geographically located in the western part of the Kłodzko Region (Fig. 1). The CO_2_-rich waters of Kudowa-Jeleniów (especially from deeper wells—K-200 and J-150) are enriched in silicon, sodium, and potassium (Table 2) if compared to waters of Polanica and Duszniki. Kiełczawa (2011) connected this with hydrolytic decay of rock-forming aluminosilicates in the crystalline basement of the Kudowa Trough (KT) . A characteristic feature of Kudowa-Jeleniów waters is also arsenic concentrations higher than in other CO_2_-rich waters of the Kłodzko Region (Ciężkowski 1990) (Table 2). The Ge–As and Ge–Fe correlations (Fig. 7, Online Resource 3) in Kudowa-Jeleniów waters suggest the role of a mineral co-source (likely sulphides) for arsenic, iron, and germanium.

Relations between germanium-to-X_element_ (Si, As, Fe, Zn) mass ratios in groundwater normalized for mean composition of the upper crust rocks (see Methods) are given in Online Resources 5–10 and are helpful in indicating the origin of germanium and other elements considered. Quasi-constant values of a Ge:X_element_ ratios indicate that: (1) two non-germanium elements (shown on particular graph) originate from different source mineral phases, and (2) germanium (principally) comes from the same phase as the second chemical element (included in a given parameter). On the other hand, directly proportional relationship between Ge:X_element_ ratios testifies that: (1) both non-germanium elements likely derive from the same phase(s), and (2) germanium might originate from the same phase(s) as (both?) non-germanium elements.

Observed ratio patterns might be also affected by immobilization of element(s) by sink phases and/or be produced by dynamic equilibrium between phases and/or processes responsible for release to and immobilization of the elements from the solution.

*Thermal waters of Lądek and Cieplice*. The aquifer rocks of Lądek thermal waters are Proterozoic gneisses of Gierałtów (Ciężkowski et al. 2011). Generally, their mineral composition consists of primary (quartz, plagioclases (Ol-Ab), K-feldspars), secondary (muscovite-sericite, biotite, chlorites, Fe oxides), and accessory (garnets, zircons, titanite, apatites, epidotes) minerals (Butkiewicz 1968; Smulikowski 1979).

The thermal waters of Cieplice occur in granitoides of the Karkonosze massif (Dowgiałło and Fistek 1995; Fistek and Fistek 2005). The Karkonosze pluton consists of several granite facies (equigranular and porphyritic) and many hybrid facies (hybrid diorite-granodiorite, microgranular mafic enclaves, composite dykes, late mafic dykes) (Słaby and Martin 2008). Granitoides are mainly composed of K-feldspars, plagioclases, quartz, biotites, and muscovites, and in hybrid rocks and lamprohyres also occur amphiboles, mainly hornblende; and accessory apatite, zircon, allanite, titanite, epidote, magnetite, ilmenite, and monazite (Borkowska 1966). Small ore deposits and ore occurrences of numerous elements: Fe, Cu, Sn, As, U, Co, Au, Ag, Pb, Ni, Bi, Zn, Sb, Se, S, Th, REE, Mo, W, and Hg are scattered within granite, granite-related pegmatites, contact aureole of the granite, and within the metamorphic envelope (Mochnacka et al. 2015).

Based on Ge/X_element_ ratio patterns (Online Resources 5, 6, 7), the basic source phases for germanium in Lądek and Cieplice thermal water systems appear to be silicate minerals. The Ge- and As- co-bearing minerals (likely sulphides) are also less probable as germanium sources (Online Resources 8, 9). However, the role of As-bearing sulphides is probably less significant due to their occurrence in trace amounts and significant dispersion. Zn and Fe minerals seem not to be significant as a potential source of germanium in either thermal water systems.

*CO*_*2*_*-rich waters in the western part of the Kłodzko Region (Kudowa, Jeleniów, Duszniki, Polanica—KJDP)*. Interpretation of Ge:X_element_ ratio patterns (Online Resources 5, 8, 9) leads to the conclusion that increased germanium concentrations (especially in Kudowa and Jeleniów waters) are likely brought on by the same processes which are responsible for releasing arsenic into solution. The role of silicates as a source of germanium is seemingly less important (Online Resource 7) than arsenic-containing phases (Online Resource 5) and might be effectively masked by iron-bearing solids (Online Resource 6). The CO_2_-rich mineral waters of this region are usually rich in iron. A relationship between zinc and germanium is not evident (Online Resource 10).

The mineral waters of Kudowa and Jeleniów occur in sedimentary Upper Cretaceous rocks (mainly sandstones and mudstones) in a structure named the Kudowa Trough (KT). The KT is the eastern part of the Nachod Basin and is filled by Permian, Cretaceous (conglomerates, sandstones, shales, marls), and Neogene deposits. The basement and surroundings of KT are built of Early Paleozoic metamorphic rocks (mainly of schists, phyllites, and amphibolites of Stronie and Nové Mĕsto formations) and Carboniferous granitoids (Kudowa-Olešnice granitoids from E, Nový Hrádek granitoids from SW). Kudowa-Olešnice granitoids were generated from metamorphic rocks (mainly schists) and show a clear geochemical affinity to the metamorphic schists of the Stronie Formation (Bachliński 2002). Both granitoids and their metamorphic source rocks are very poor in ore minerals. There are no reports of the presence of arsenic sulphides or arsenic-bearing sulphide minerals in the above-mentioned rocks. Among the accessory minerals (apatites, titanite, epidote, zircon, sillimanite, leucoxene, and hematite) which are present in Kudowa-Olešnice granitoids (Borkowska 1959; Bachliński 2002) the only epidote and leucoxene usually contain increased germanium content (Ivanov 1996). Moreover, the mentioned accessory minerals do not explain the presence of arsenic in Kudowa-Jelenów waters.

The occurrence of CO_2_-rich KJDP waters is associated with the Poříčí-Hronov fault zone and accompanying dislocations. The CO_2_-rich mineral waters enriched in arsenic are also known in the localities of Běloves, Nachod, and Hronov in the Czech part of the Nachod Basin, where are also related to Poříčí-Hronov and associated dislocations (Jetel and Rybářová 1979). The presence of germanium has not been studied in the CO_2_-rich waters of the Czech part of the Nachod Basin yet.

At the outflows, exploited CO_2_-rich waters of the KJDP area are nominally cold (*T* < 20 °C). However, occurrence of thermal CO_2_-rich waters in this part of the Sudetes is proven by findings in Jeleniów (P-5 well, 133 m deep, *T* = 20.5 °C), Duszniki (GT-1 well, 1695 m deep, *T* = 34.7 °C), and Batňovice (BA-1 well, 1324 m deep, *T* = 32 °C) (Jetel and Rybářová 1979; Dowgiałło 1987; Dowgiałło and Fistek 2003). Local high heat flow in Sudetes has been explained by mid-to-late Cenozoic volcanism and radiogenic heat (Dowgiałło 2002).

The absence of sufficient geochemical and mineralogical research in both Polish and Czech parts of the Nachod Basin area causes that the origin of enrichment of Kudowa-Jeleniów’s mineral waters in arsenic and germanium is still an intriguing and open issue. The current lack of information on As-bearing sulphides in bedrocks enveloping the KT forces to seek other presumptive explanations. This might be arsenic and germanium source(s) in deeper, poorly studied crystalline bedrocks and/or migration of substances of post-magmatic origin along deep-seated dislocations related to the seismically active (Zedník and Pazdírková 2010; Kolínský et al. 2012) Poříčí-Hronov fault zone.

The characteristic feature of *CO*_*2*_-*rich waters of Szczawno and Długopole* is enrichment in iron, which is greater in Długopole waters (Table 2). The curative waters of Szczawno discharge along the tectonic zone, from Lower Carboniferous sedimentary rocks (mainly greywacke sandstones, with conglomerates and mudstones) (Ciężkowski 1990). The mineral composition of pebbles (e.g. quartzites, diabases, greenschists, schists, porphyries, keratophyres, phyllites), matrix, and/or cement (clayey, ferruginous, siliceous) of clastic aquifer bedrocks is very varied (Bossowski and Czerski 1988). The curative waters of Długopole are likely related to Proterozoic mica schists (Ciężkowski 1990). In mica schists, apart from main rock-forming minerals (biotite, muscovite-sericite, acid plagioclases, K-feldspars—usually microcline, and quartz), chlorites, epidote, quite common garnets, and much less abundant staurolite and kyanite also occur in minor quantities (Dumicz 1964).

In the curative waters of Szczawno and Długopole, a visible relationship between germanium and iron (Online Resources 8, 10) and between germanium and silicon (Online Resource 7) can be found, which suggests the important role of Fe-bearing silicates in releasing the elements into solution. Most likely, in the case of both Szczawno and Długopole curative waters, the most probable and important source of iron and germanium is biotite.

*CO*_*2*_-*rich waters of Świeradów and Czerniawa* are related to the granitogneisses, gneisses, and mica schists (Ciężkowski 1983) of the Izera Mountains. In these waters, the relationship between germanium and iron (Online Resources 6, 8, 10) is apparently revealed. Most of Świeradów-Czerniawa curative waters are very rich in iron (Table 2). The iron-bearing minerals (biotites, pyrites, Fe oxides; Szałamacha and Szałamacha 1968; Smulikowski 1972) are the likely source phases for germanium in solution.

The studied CO_2_-rich waters in the Sudetes are modern, tritium-bearing waters (Ciężkowski 1990) of renewable resources with short turnover times in hydrogeological systems. In such conditions, aqueous chemistry is mainly governed by the kinetics of water–rock processes. The thermal waters of Lądek and Cieplice also represent renewable resources. However, due to their much longer ages (estimated Lądek waters age—from several to 20 ka; Cieplice waters—approx. 20–30 ka; e.g. Ciężkowski 1990) and much deeper circulation in the systems, both thermal waters could achieve chemical equilibrium with respect to rock-forming silicate minerals in deep parts of the systems as was indicated by multi-component chemical geotermometer calculations (Leśniak and Nowak 1993; Dobrzyński and Leśniak 2010).

The above-mentioned differences are also noticeable in terms of germanium and related solutes. Germanium (and probably also silicon) concentrations in thermal waters are controlled by the solubility of rock-forming silicate minerals, conceivably by neo-formed vein quartz. Variable germanium concentrations in cold CO_2_-rich waters appear to be due to the kinetics of mineral decay. This gives hope of finding waters richer in germanium than have previously been found. At present, the water richest in germanium known in the Sudetes is curative mineral water from the K-200 well (in Kudowa Spa). There is a chance of finding other Sudetes mineral waters enriched in germanium. The most promising seem to be water-bearing zones related to the Poříčí-Hronov fault zone and accompanying dislocations in the crystalline basement of the KT (Poland) and in Czech part of the Nachod Basin.

Finding a new germanium-rich groundwater requires more detailed geochemical research of water–rock systems in the Sudetes. There is little recognition of germanium bedrock and aqueous geochemistry in the Sudetes area. Data on germanium content in aquifer bedrocks are rare, as for Izera gneisees (mean 1.9 ppm of Ge in gneisses of the Izera Mountains; Oberc-Dziedzic et al. 2005).

## Perspectives, limitations and requirements of using waters enriched in germanium in balneotherapy

Recently, high concentrations of germanium (up to 36 µg/L) have been found in low-temperature (12.3 °C) mineral CO_2_-rich water in the Bieszczady Mountains (Carpathians Mountains, SE Poland) in sedimentary (mainly sandstones and conglomerates of Cretaceous-Paleocene) aquifer (Dobrzyński et al. 2011). Finding more groundwaters enriched in germanium in the Carpathians and the Sudetes is probably a question of time. The studies carried out suggest the possibility of finding germanium-rich waters in the westernmost part of the Kłodzko Region, especially in the vicinity of Kudowa and Jeleniów.

The positive correlation between germanium and arsenic found in Kudowa-Jeleniów waters raises the concern that waters enriched in germanium might not meet standards in terms of arsenic. The maximum acceptable concentration (MAC) of As in bottled water is 10 µg/L (Order 2011). However, waters containing an arsenic concentration above 10 µg/L might be used as curative waters. The MAC of As is not established if the curative water is used for creno-therapeutical cure (treatment of disease by drinking mineral water) for a period shorter than one month. In the case of therapy longer than 1 month, the MAC of arsenic is 50 and 100 µg/L, for drinking therapy and inhalation, respectively (Order 2006).

Germanium seems to be a very promising microelement that could be used as a prophylactic and for therapy of such diseases as cancer, HIV infection, autoimmune diseases, arthritis, or senile osteoporosis (e.g. Hirayama et al. 2003; Goodman 1988b; Fujii et al. 1993; Seaborn and Nielsen 1994). Therefore, there is a strong need for further and more detailed research on the mechanism of germanium action. The most important topics to investigate are the safe and effective doses as well as the duration of treatment. Due to a specific tendency of germanium to accumulate in the organs and tissues and its relatively long half-life, sanatorium treatment might have two essential advantages: 2 or 3 weeks of stationary therapy (treatment in sanatorium or spa hotels, when the curation for all the time is supervised by a doctor) would be long enough to exert therapeutic effects on patients, at the same time be short enough to prevent any side effects connected with accumulative toxicity of germanium. As retention of the element in the body is quite long, the positive effects of the therapy could be observed even for a long time after the sanatorium treatment. Moreover, the patient could be monitored by a specialist during the whole therapy and any problems related to side effects could be immediately resolved.

## Conclusions

The studies carried out show that the greatest chance for finding water enriched in germanium in the Sudetes Mountains (SW Poland) is in CO_2_-rich water system in the westernmost part of the Kłodzko region, especially in the crystalline basement of Kudowa Trough (eastern part of transboundary Nachod Basin).

Relationships between chemical elements suggest that germanium comes from the same source phase(s) as arsenic which might be sulphide minerals. In the absence of a sufficient geochemical and mineralogical recognition, it might only be hypothesized that the delivery of germanium to CO_2_-rich mineral waters in this area would is likely to arise from deeper, crystalline bedrocks and/or migration of substances of post-magmatic origin along deep-seated dislocations related to the seismically active Poříčí-Hronov fault zone. An explanation of germanium origin in the mineral/curative waters of the Kudowa Trough area requires further study on the bedrock geochemistry and mineralogy, and also on the geochemistry of CO_2_-rich waters associated with the Poříčí-Hronov fault zone and accompanying dislocations, including waters in the Czech part of Nachod Basin.

Germanium in other studied CO_2_-rich waters seems to be the result of a kinetic process between aquifer minerals and groundwater under short turnover time in hydrogeological systems. In these aquifer systems, iron-bearing solid phases (silicates, oxides) probably play an important role in the release of Ge into solution.

Germanium in Cieplice and Lądek thermal waters confirms silicon-like geochemistry, and reveals a strong origin from silicate minerals. This seems to be more likely than germanium originating from highly dispersed in crystalline (granite, granite gneiss) bedrocks of arsenic-containing sulphides. Confirmation of the probable role of vein quartz in controlling the Ge–Si relation requires research on germanium in neo-formed silica solids. Recent findings in the Jelenia Góra geothermal system seem to confirm the role of secondary silica forms in controlling germanium solute (Dobrzyński et al. 2017). It should be noted that the geochemistry of germanium in groundwater, especially in fresh and mineral waters beyond the areas of thermal waters, is still poorly examined and understood.

Inducing interest in the use of germanium in biology and medicine will also conceivably entail the applications of germanium-rich groundwater in balneotherapy. Promising results on the bioavailability and the biochemical role of germanium seem to encourage research using natural water enriched in germanium, as with some of the curative waters in the existing health resorts. The advantage of such research would enable further biomedical research in the medical facilities of sanatoriums and spa hospitals in the use of germanium-rich waters.

## Electronic supplementary material

Below is the link to the electronic supplementary material.

**Online Resource 1 (ESM_1)** Germanium concentration versus ionic strength of groundwater. Intake numbers as in Table 2 (TIFF 8507 kb)

**Online Resource 2 (ESM_2)** Germanium versus bicarbonates in groundwater. Intake numbers as in Table 2 (TIFF 8507 kb)

**Online Resource 3 (ESM_3)** Germanium versus iron in groundwater. Intake numbers as in Table 2 (TIFF 8507 kb)

**Online Resource 4 (ESM_4)** Germanium versus zinc in groundwater. Intake numbers as in Table 2 (TIFF 8507 kb)

**Online Resource 5 (ESM_5)** The Ge:As versus Ge:Si mass ratios in groundwater normalized with respect to the upper crust composition. Intake numbers as in Table 2 (TIFF 8507 kb)

**Online Resource 6 (ESM_6)** The Ge:Si versus Ge:Fe mass ratios in groundwater normalized with respect to the upper crust composition. Intake numbers as in Table 2 (TIFF 8507 kb)

**Online Resource 7 (ESM_7)** The Ge:Si versus Ge:Zn mass ratios in groundwater normalized with respect to the upper crust composition. Intake numbers as in Table 2 (TIFF 8507 kb)

**Online Resource 8 (ESM_8)** The Ge:As versus Ge:Fe mass ratios in groundwater normalized with respect to the upper crust composition. Intake numbers as in Table 2 (TIFF 8507 kb)

**Online Resource 9 (ESM_9)** The Ge:As versus Ge:Zn mass ratios in groundwater normalized with respect to the upper crust composition. Intake numbers as in Table 2 (TIFF 8507 kb)

**Online Resource 10 (ESM_10)** The Ge:Fe versus Ge:Zn mass ratios in groundwater normalized with respect to the upper crust composition. Intake numbers as in Table 2 (TIFF 8507 kb)

## References

[CR1] Arnórsson S (1984). Germanium in Icelandic geothermal systems. Geochimica et Cosmochimica Acta.

[CR2] Asai K (1981). Miracle cure organic germanium.

[CR3] Asaka T, Nitta E, Makifuchi T, Shibazaki Y, Kitamura Y, Ohara H, Matsushita K, Takamori M, Takahashi Y, Genda A (1995). Germanium intoxication with sensory ataxia. Journal of the Neurological Sciences.

[CR4] Aso H, Kobayashi H, Sugiyama K, Saito K, Chiyotani K, Ebina T, Ishida N (1988). Induction of interferon and activation of NK cell and macrophages in pneumoconiotic patients after oral administration of Ge-132 (2-carboxyethylgermanium sesquioxide). The Journal of Japan Accident Medical Association.

[CR5] Bachliński, R. (2002). *Petrological, geochemical, and geochronological studies of crystalline rocks from the vicinity of Kudowa*. Ph.D. thesis, Institute of Geological Sciences, Warsaw. (**in Polish**).

[CR6] Bardet J (1914). Extraction du germanium des eaux de Vichy. Comptes rendus de l’Académie des sciences.

[CR7] Bernstein L (1985). Germanium geochemistry and mineralogy. Geochimica et Cosmochimica Acta.

[CR8] Bitschene, P., & Sessler, W. (2012). Gerolsteiner Brunnen, Nürburg Quelle und Dauner Sprudel. Herkunft und Besonderheiten dieser Mineralwässer. *Landkreis Vulkaneifel Heimatjahrbuch 2012* (pp. 64–71), Monschau.

[CR9] Borkowska M (1959). On the granitoids of Kudowa, as compared with the main types of the acid intrusions of the Sudeten Mts. and the Sudetic Foreland. Archiwum Mineralogiczne.

[CR10] Borkowska M (1966). Petrography of the Karkonosze granite. Geologia Sudetica.

[CR11] Bossowski A, Czerski M (1988). Explanations to the detailed geological map of the Sudetes at the scale 1:25 000, the Boguszów sheet.

[CR12] Butkiewicz T (1968). Crystalline schists in the Krowiarki range of the Kłodzko Mts. Geologia Sudetica.

[CR13] Ciężkowski W (1983). Hydrogeological unit of the Góry Izerskie carbon dioxide waters. Geological Quarterly.

[CR14] Ciężkowski W (1990). A study on the hydrogeochemistry of curative waters in the Polish Sudetes Mountains (SW Poland). Prace Naukowe Instytutu Geotechniki Politechniki Wrocławskiej.

[CR15] Ciężkowski W, Liber-Makowska E, Ciekot B, Ogórek A (2011). Characteristics of the conditions and exploitation of thermal waters in Lądek-Zdrój. Technika Poszukiwań Geologicznych.

[CR16] COT. (2008). *Committee on Toxicity statement on the 2006 UK total diet study of metals and other elements*. https://cot.food.gov.uk/sites/default/files/cot/cotstatementtds200808.pdf. Accessed January 19, 2017.

[CR17] Criaud A, Fouillac AM (1986). Etude des eaux thermominerales carbogazeuses du Massif Central Francais. II. Comportement de quelques metaux en trace, de l’arsenic, de l’antimoine et du germanium. Geochimica et Cosmochimica Acta.

[CR18] Dhingra HM, Umsawasdi T, Chiuten DF, Murphy WK, Holoye PY, Spitzer G, Valdivieso M (1986). Phase II study of spirogermanium in advanced (extensive) non-small cell lung cancer. Cancer Treatment Reports.

[CR19] Dobrzyński D, Gruszczyński T, Birski Ł (2017). Germanium as an indicator of hydrogeochemical conditions in the Jelenia Góra geothermal system. Przegląd Geologiczny (Polish Geological Review).

[CR20] Dobrzyński D, Leśniak PM, Kania J, Kmiecik E, Zuber A (2010). Two contrasting geothermal systems—Towards the identification of geochemical reaction pattern and groundwater temperature, the Sudetes, Poland. 38th IAH congress, “Groundwater quality sustainability” Krakow. Extended abstracts book.

[CR21] Dobrzyński, D., Słaby, E., & Mętlak, A. (2011). Germanium geochemistry in mineral groundwater from mountain areas of Southern Poland—A case study of its affinity to other elements. In *Geological and medical sciences for a safer environment*, *Book of Abstracts. GeoMed2011* (pp. 185–185). Bari, Italy.

[CR22] Dowgiałło J (1987). Geohydrothermal problems of the Sudety region. Przegląd Geologiczny (Polish Geological Review).

[CR23] Dowgiałło J (2002). The Sudetic geothermal region of Poland. Geothermics.

[CR24] Dowgiałło J, Fistek J (1995). The Jelenia Góra geothermal system (Western Sudetes, Poland). Bulletin of the Polish Academy of Sciences, Earth sciences.

[CR25] Dowgiałło J, Fistek J (2003). New findings in the Walbrzych–Kłodzko geothermal sub-region (Sudetes, Poland). Geothermics.

[CR26] Duane MJ, Pigozzi G, Harris C (1997). Geochemistry of some deep gold mine waters from the western portion of the Witwatersrand Basin, South Africa. Journal of African Earth Sciences.

[CR27] Dumicz M (1964). Geology of the crystalline massif of the Bystrzyckie Mts. Geologia Sudetica.

[CR28] El Wardani SA (1957). On the geochemistry of germanium. Geochimica et Cosmochimica Acta.

[CR29] Elmi SA (2009). Gallium and germanium distribution in geothermal water. Geothermal Training Programme, Reports.

[CR30] Enghag, P. (2004). Germanium. In *Encyclopedia of the elements* (pp. 923–933). Wiley, Hoboken.

[CR31] Evans MJ, Derry LA (2002). Quartz control of high germanium-silicon ratios in geothermal waters. Geology.

[CR32] Faloona GR, Levine SA (1988). The use of organic germanium in chronic Epstein-Barr Virus Syndrome (CEBVS). Journal of Orthomolecular Medicine.

[CR33] Fistek J, Fistek A, Mierzejewski MP (2005). Thermal waters in the Polish part of the Karkonosze Massif. Karkonosze. Przyroda nieożywiona i człowiek.

[CR34] Frengstad B, Midtgård AK, Banks D, Krog JR, Siewers U (2000). The chemistry of Norwegian groundwaters: III. The distribution of trace elements in 476 crystalline bedrock groundwaters, as analysed by ICP-MS techniques. Science of the Total Environment.

[CR35] Frenzel M, Ketris MP, Gutzmer J (2014). On the geological availability of germanium. Mineralium Deposita.

[CR36] Fujii A, Kuboyama N, Yamane J, Nakao S, Furukawa Y (1993). Effect of organic germanium compound (Ge-132) on experimental osteoporosis in rats. General Pharmacology: The Vascular System.

[CR37] Furst A (1987). Biological testing of germanium. Toxicology and Industrial Health.

[CR38] Gerber GB, Leonard A (1997). Mutagenicity, carcinogenicity and teratogenicity of germanium compounds. Mutation Research.

[CR39] Gijbels, R., Van Grieken, R., Blommaert, W., Vandelannoote, R., Van’t Dack, L., Van Espen, P., et al. (1983). *Application of analytical methods for trace elements in geothermal waters*. *Part II: Plombières, Bains-les-Bains, Bourbonne (Vosges)* (pp. 1–149). Commission of the European Communities, Final Report EEC-Contract 119-76-EGB.

[CR40] Goldstein F (1927). Über den Einfluss des Wassers aus dem Kreise Daun in der Eifel auf den Krebs. Journal of Cancer Research and Clinical Oncology.

[CR41] Goldstein F (1932). Der Krebs. Seine Verhütung und Heilung nach den Grundsätzen moderner Säftelehre.

[CR42] Goleva GA, Vorobjeva IN (1967). Some features of germanium migration in underground waters of ore deposits. Geochemistry International (Geokhimiya).

[CR43] Goodman S (1988). Germanium. The health and life enhancer.

[CR44] Goodman S (1988). Therapeutic effects of organic germanium. Medical Hypotheses.

[CR45] Harvey J, McFadden M, Smith FP, Joubert L, Schein PS (1990). Phase I study of oral spirogermanium. Investigational New Drugs.

[CR46] Hirayama C, Suzuki H, Ito M, Okumura M, Oda T (2003). Propagermanium: a nonspecific immune modulator for chronic hepatitis B. Journal of Gastroenterology.

[CR47] Hirono M, Yoshihara T, Suzuki M (1991). The therapeutic effect of 2-carboxyethylgermanium sesquioxide (Ge-132) in the treatment of gynecologic malignant diseases. Biotherapy.

[CR48] Höll R, Kling M, Schroll E (2007). Metallogenesis of germanium—A review. Ore Geology Reviews.

[CR49] Ivanov VV, Burienkov EK (1996). Rare p-elements. Ecological geochemistry of elements—6 volumes set.

[CR50] Jetel J, Rybářová L (1979). Minerální vody Východočeského kraje.

[CR51] Kamen B (1987). Germanium. A new approach to immunity.

[CR52] Kang KS, Yun JW, Yoon B, Lim YK, Lee YS (2001). Preventive effect of germanium dioxide on the inhibition of gap junctional intercellular communication by TPA. Cancer Letters.

[CR53] Kawakami H, Nozaki H, Koga A (1956). Chemical studies on the hot Springs of Beppu. I. Trace elements in the hot springs of Beppu. (2). Distribution of arsenic. Nippon Kagaku Zassi (Journal of the Chemical Society of Japan).

[CR54] Kenison Falkner K, Church M, Measures CI, LeBaron G, Thouron D, Jeandel C, Stordal MC, Gill GA, Mortlock R, Froelich P, Chan L-H (1997). Minor and trace element chemistry of Lake Baikal, its tributaries, and surrounding hot springs. Limnology and Oceanography.

[CR55] Kiełczawa B (2011). The main hydrogeochemical processes affecting the composition of certain naturally carbonated waters of southwestern Poland. Geological Quarterly.

[CR56] Kobayashi A, Ogra Y (2009). Metabolism of tellurium, antimony and germanium simultaneously administered to rats. The Journal of Toxicological Sciences.

[CR57] Koga A (1967). Germanium, molybdenum, copper and zinc in New Zealand thermal waters. New Zealand Journal of Science.

[CR58] Kolínský P, Valenta J, Gaždová R (2012). Seismicity, groundwater level variations and earth tides in the Hronov-Porici fault zone, Czech Republic. Acta Geodynamica et Geomaterialia.

[CR59] Konno K, Motomiya M, Kotaro O, Nakai Y, Nagahama F, Tanabe T, Suzuki A, Nakabayashi T (1990). Results of multicenter placebo-controlled study on organogermanium compound in the treatment of unresectable lung cancer. Biotherapy.

[CR60] Kraynov SR (1967). Geochemistry of germanium in carbonate thermal waters (exemplified by the Great Causasus and the Pamir). Geochemistry International (Geokhimiya).

[CR61] Kuroda K (1939). The occurrence of germanium in the hot springs of Senami. Bulletin of the Chemical Society of Japan.

[CR62] Kurtz AC, Derry LA, Chadwick OA (2002). Germanium–silicon fractionation in the weathering environment. Geochimica et Cosmochimica Acta.

[CR63] Leśniak PM, Nowak D (1993). Water-rock interaction in some mineral waters of the Sudetes, Poland: Implications for chemical geothermometry. Annales Societatis Geologorum Poloniae.

[CR64] Levine SA (1987). Organic germanium. A novel dramatic immunostimulant. Journal of Orthomolecular Medicine.

[CR65] Lewis BL, Andreae MO, Froelich PN, Mortlock RA (1988). A review of the biogeochemistry of germanium in natural waters. The Science of the Total Environment.

[CR66] Lin CH, Chen SS, Lin YC, Lee YS, Chen TJ (2006). Germanium dioxide induces mitochondria-mediated apoptosis in Neuro-2A cells. Neurotoxicology.

[CR67] MAFF. (1997). *1994 total diet study: Metals and other elements*. London: United Kingdom Ministry of Agriculture, Food and Fisheries, Food Standards Agency. Food Surveillance Information Sheet No. 131.

[CR68] Mochnacka K, Oberc-Dziedzic T, Mayer W, Pieczka A (2015). Ore mineralization related to geological evolution of the Karkonosze-Izera Massif (the Sudetes, Poland)—Towards a model. Ore Geology Reviews.

[CR69] Nagahama F, Anso T, Ito R, Tanabe K, Sakai I (1986). The clinical study on the preventive effects of Ge-132 against the upper airway infectious syndrome of pneumoconiosis patients—double blind test. The Journal of Japan Accident Medical Association.

[CR70] Nagata N, Yoneyama T, Yanagida K, Ushio K, Yanagihara S, Matsubara O, Eishi Y (1985). Accumulation of germanium in the tissues of a long-term user of germanium preparation died of acute renal failure. The Journal of Toxicological Sciences.

[CR71] Nakata Y, Niahii K, Yonei T, Kawahara S, Kataoka M, Hiraki S, Takahashi I, Ohnoshi T, Kimura I (1986). Combined effect of Ge in lung cancer chemotherapy. Japanese Journal of Medicine and Pharmaceutical Science.

[CR72] Norcliffe GB (1986). Statistics for geographers.

[CR73] Nuriev AN, Lapshina NF, Dzhabbarova ZA (1968). Germanium in the stratal waters, oils and rocks of oil deposits. Geochemistry International (Geokhimiya).

[CR74] Oberc-Dziedzic T, Pin C, Kryza R (2005). Early Palaeozoic crustal melting in an extensional setting: petrological and Sm–Nd evidence from the Izera granite-gneisses, Polish Sudetes. International Journal of Earth Sciences.

[CR75] Order. (2006). Order of the Minister of Health concerning the scope of the research necessary to determine the therapeutic properties of natural medicinal raw materials and healing properties of the climate, the criteria for their evaluation and a model of certificate confirming these properties. *Legislation Journal of the Republic of Poland 80*: pos. 565. (http://isap.sejm.gov.pl/DetailsServlet?id=WDU20060800565) Amended in 2016. http://isap.sejm.gov.pl/DetailsServlet?id=WDU20160001709. Accessed December 28, 2016.

[CR76] Order. (2011). Order of the Minister of Health concerning natural mineral waters, spring waters, and table waters. *Legislation Journal of the Republic of Poland 85*: pos. 466. http://isap.sejm.gov.pl/DetailsServlet?id=WDU20110850466. Accessed December 28, 2016.

[CR77] Parkhurst, D. L., & Appelo, C. A. J. (2013). Description of input and examples for PHREEQC version 3—A computer program for speciation, batch-reaction, one-dimensional transport, and inverse geochemical calculations. In *U.S. geological survey techniques and methods* (book 6, chap. A43, p. 497). http://pubs.usgs.gov/tm/06/a43. Accessed October 5, 2013.

[CR78] Peng X, Lingxia Z, Schrauzer GN, Xiong G (2000). Selenium, boron, and germanium deficiency in the etiology of Kashin-Beck disease. Biological Trace Element Research.

[CR79] Reimann C, Birke M (2010). Geochemistry of European bottled water.

[CR80] Rosenberg E (2009). Germanium: Environmental occurrence, importance and speciation. Reviews in Environmental Science & Biotechnology.

[CR81] Rosenfeld G (1954). Studies of the metabolism of germanium. Archives of Biochemistry and Biophysics.

[CR82] Rudnick RL, Gao S, Rudnick RL (2003). Composition of the continental crust. Treatise on geochemistry, volume 3—The crust.

[CR83] Saiers JH, Slavik M, Stephens RL, Crawford ED (1987). Therapy for advanced renal cell cancer with spirogermanium: A southwest oncology group study. Cancer Treatment Reports.

[CR84] Satgé J (2004). Some applications of germanium and its derivatives. Main Group Metal Chemistry.

[CR85] Schauss AG (1991). Nephrotoxicity and neurotoxicity in humans from organogermanium compounds and germanium dioxide. Biological Trace Element Research.

[CR86] Schauss AG (1991). Nephrotoxicity in humans by the ultratrace element germanium. Renal Failure.

[CR87] Seaborn CD, Nielsen FH (1994). Effects of germanium and silicon on bone mineralization. Biological Trace Element Research.

[CR88] Sellappa S, Jeyaraman V (2011). Antibacterial properties of organic germanium against some human pathogens. International Journal of Pharma and Bio Sciences.

[CR101] Słaby E, Martin H (2008). Mafic and felsic magma interaction in granites: The Hercynian Karkonosze Pluton (Sudetes, Bohemian Massif). Journal of Petrology.

[CR89] Smulikowski W (1972). Petrogenetic and structural problems of the northern cover of the Karkonosze granite. Geologia Sudetica.

[CR90] Smulikowski K (1979). Polymetamorphic evolution of the crystalline complex of Śnieżnik and Góry Złote Mts in the Sudetes. Geologia Sudetica.

[CR91] Staufer M (1985). Germanium in heilenden Wässern. Ärztezeitschrift für Naturheilverfahren.

[CR92] Szałamacha J, Szałamacha M (1968). The metamorphic series of the Karkonosze—Góry Izerskie Mountainous Block. Biuletyn Państwowego Instytutu Geologicznego.

[CR93] Tanaka N, Ohida J, Ono M, Yoshiwara H, Beika T, Terasawa A, Yamada J, Morioka S, Mannami T, Orita K (1984). Augmentation of NK activity in peripheral blood lymphocytes of cancer patients by intermittent Ge-132 administration. Japanese Journal of Cancer & Chemotherapy.

[CR94] Tao S-H, Bolger PM (1997). Hazard assessment of germanium supplements. Regulatory Toxicology and Pharmacology.

[CR95] Tziritis E, Kelepertzis A, Lambrakis N (2011). Trace and ultra-trace element hydrochemistry of Lesvos thermal springs. Advances in the research of aquatic environment.

[CR96] Uzumasa Y, Nasu Y, Toshiko S (1959). Chemical investigations of hot springs in Japan: XLIX. Germanium contents of hot springs. Nippon Kagaku Zassi (Journal of the Chemical Society of Japan).

[CR97] Van der Spoel JI, Stricker BH, Esseveld MR, Schipper ME (1990). Dangers of dietary germanium supplements. Lancet.

[CR98] WHO. (2008). *Guidelines for drinking*-*water quality. Third edition incorporating the first and second addenda* (Vol. 1), *Recommendations*. Geneva: WHO. http://www.who.int/water_sanitation_health/dwq/gdwq3rev/en/. Accessed July 20, 2014.

[CR99] Wood SA, Samson IM (2006). The aqueous geochemistry of gallium, germanium, indium and scandium. Ore Geology Reviews.

[CR100] Zedník J, Pazdírková J (2010). Seismic activity in the Czech Republic in 2008. Studia Geophysica et Geodaetica.

